# Painful pachydermodactyly in a 39-year-old woman: A case report and review of the literature

**DOI:** 10.1016/j.amsu.2021.102871

**Published:** 2021-09-17

**Authors:** Salman Hussain, Maryam Ehtesham, Talal Almas, Ali Aldei

**Affiliations:** a RCSI University of Medicine and Health Sciences, Dublin, Ireland; b Department of Rheumatology, Al-Amiri Hospital, Kuwait

**Keywords:** Pachydermodactyly, Digital fibromatosis, Painless joint swelling, Proximal interphalangeal joint, Distal interphalangeal joint

## Abstract

We chronicle the case of a 39-year-old female who presented to the rheumatology clinic with a history of chronic, symmetrical polyarticular pain in her hands. Meticulous diagnostic workup to exclude ubiquitous culprit aetiologies, such as rheumatoid arthritis and psoriatic arthritis, was performed. A detailed clinical examination was performed and, coupled with the radiological imaging findings, divulged an underlying diagnosis of pachydermodactyly. The patient was commenced on etoricoxib to alleviate the pain, and was advised to avoid repetitive trauma to her hands. The present report delineates a unique case of painful pachydermodactyly, affecting both proximal interphalangeal joints as well as distal interphalangeal joints. To the best of our knowledge, this is the first case from the state of Kuwait. We further review the literature in order to better elucidate the varying clinical manifestations of an elusive and rare rheumatological condition.

## Highlights

• Pachydermodactyly is a rare disease that can be easily misdiagnosed and treated as an inflammatory arthritis leading to the initiation of unnecessary systemic therapies.
• Pachydermodactyly follows a benign course, therefore aggressive therapy is typically not warranted.
• Pachydermodactyly presents a diagnostic challenge due to its ability to mimic several other etiologies that render the same physical finding of progressive proximal interphalangeal joint swelling.

## 1 Introduction

Pachydermodactyly (PDD) is a rare, benign form of digital fibromatosis that is characterized clinically by asymptomatic, progressive soft tissue swelling and thickening of the periarticular skin, primarily affecting the lateral aspects of the proximal interphalangeal (PIP) joints of the fingers [1,2]. It has been reported to have a predilection for affecting adolescent males with a male-to-female ratio of 3.9:1. While the exact cause remains unknown and the etiology is not completely clear, it is thought to occur due to the excessive mechanical manipulation of PIP joints [3]. PDD has also been associated with obsessive-compulsive disorder (OCD) and generalized anxiety disorder (GAD) [4,5].

Due to the rarity of the disease and unfamiliarity of rheumatologists to the disease, PDD can be easily misdiagnosed and treated as an inflammatory arthritis leading to the initiation of unnecessary systemic therapies. The final diagnosis is reached upon exclusion of other causes of joint swelling, and requires a high index of suspicion particularly in patients with progressive soft tissue swelling in the absence of pain, tenderness or limited function [1]. To date, there is no widely acknowledged treatment for PDD. Nevertheless, because of the benign course of the disease, aggressive therapy is typically not warranted [6].

A younger age group of children and adolescents tend to yield a relatively higher index of suspicion for PDD. As such, the case that we delineate of a 39-year-old female offers novelty to the niche that is PDD. Given that the condition has demonstrated a greater incidence in males, our patient certainly offers a unique addition to the literature on PDD. While previously reported cases of PDD involve patients presenting with painless progressive swelling of the PIP joints, our patient presents with a unique case of painful PDD. We also reviewed the current literature and summarized previously published cases of PDD, highlighting the classic clinical and radiological findings, as well as the unusual presentations and associations with genetic conditions.

## 2 Case presentation

**Fig. 1:**
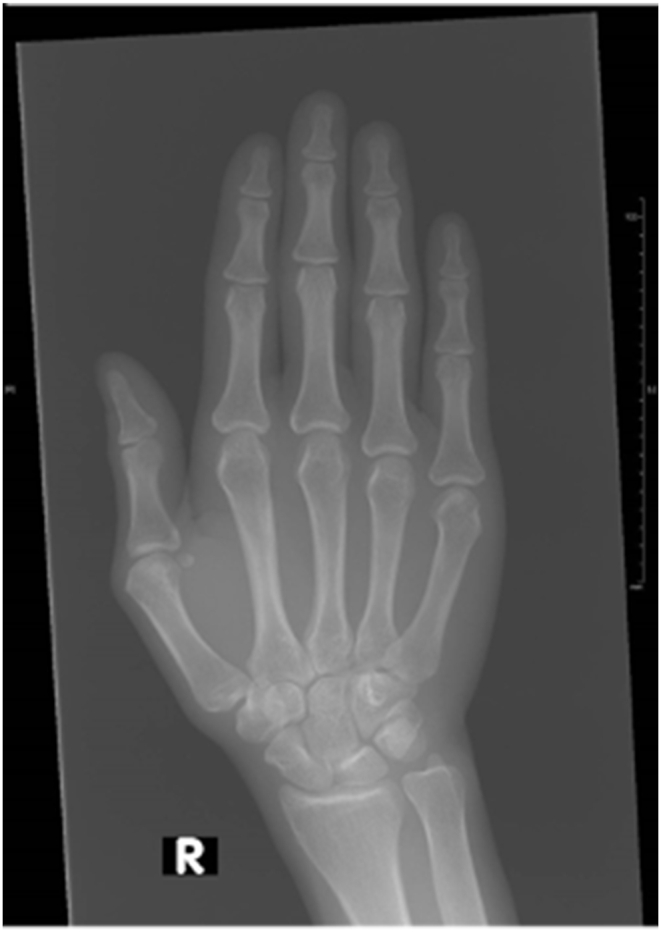
X-ray of the right hand showing only soft tissue swelling in the absence of articular or bony manifestations.

We delineate the case of a 39-year-old female who presented to the rheumatology clinic with a history of chronic, symmetrical polyarticular pain in her hands. Pertinently, she reported that the pain was particularly excruciating in her proximal interphalangeal (PIP) and distal interphalangeal (DIP) joints. The patient reported having a healthy lifestyle, with no such complaints in the past. Additionally, the patient's family history was unremarkable for any rheumatological ailments, including rheumatoid arthritis, juvenile idiopathic arthritis, or osteoarthritis. A meticulous history was obtained from the patient to better elucidate the etiology underlying her clinical presentation. She reported no history of any trauma, preceding gastrointestinal symptoms, concomitant skin lesions, or any other rheumatological afflictions. The patient also does not take any medications, a notion that precludes the potential involvement of a therapeutic regimen in the patient's current presentation. Thereafter, a thorough clinical examination was performed and turned out unremarkable. Of note, the patient did not demonstrate any skin lesions and any nail changes or rash. An examination of the patient's hands revealed no evidence of synovitis; however, an appreciable degree of tenderness was noted surrounding her PIP joints. A detailed examination of her blood parameters, including evaluation of the rheumatoid factor (RF), anti-cyclic citrullinated peptides (anti-CCP) antibodies, and anti-nuclear antibodies (ANA) was thus performed and turned out unremarkable. Interestingly, her vitamin D levels were noted to hover below the normal range. Thereafter, a radiograph of her hands was ordered and divulged the findings delineated in Fig. 1 below.

Given the patient's clinical history, physical examination, and radiological examinations, a diagnosis of pachydermodactyly was considered plausible. In order to curb the severity of the patient's worsening clinical symptoms, a vitamin D 50,000 IU per oral once weekly regimen was commenced for a total duration of eight weeks followed by a once weekly regimen for the next six months. In addition to this, etoricoxib 90 mg per oral once daily regimen was instituted for 14 days. In the aftermath of treatment with the aforementioned therapeutic regimen, the patient's clinical symptoms promptly abated. The patient continues to do well to date with no further complaints necessitating rheumatology consultations.

## 3 Methods

**Fig. 2:**
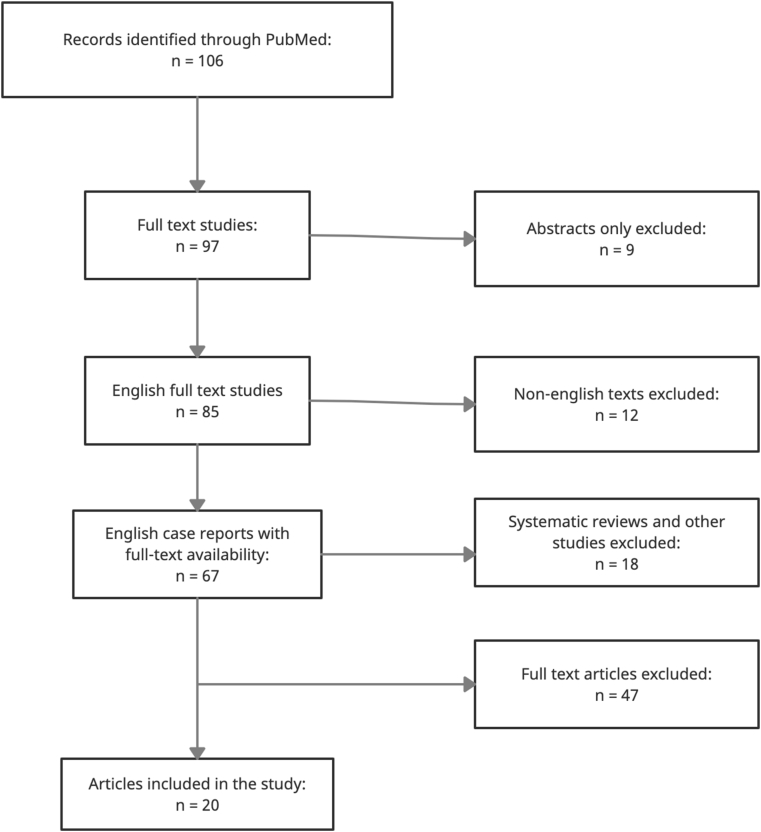
Flowchart of systematic literature review.

In order to better understand the exceedingly rare condition, we queried the Pubmed database using the term “Pachydermodactyly”. The search resulted in a total of 106 papers. Of these, we excluded papers that were not in the English language, abstract-only papers, systematic reviews and other studies, leaving behind case reports. After excluding 9 abstract only papers, 97 full text papers were identified. 12 papers of these full text studies were not in English, resulting in 85 papers. 18 papers that were not case reports were excluded. Finally 67 case reports that were in the English language and had full text availability were identified. Careful perusal of the 67 studies, 47 were excluded and 20 quality case reports were ultimately included in our study (Table 1). The results of the systematic literature review are documented in the flow chart below (Fig. 2).

**Table 1:** A detailed elucidation of the literature review performed in order to better understand the clinical manifestations, diagnostic modalities, and treatment strategies for pachydermodactyly [[1], [2], [3], [4], [5], [6], [7], [8], [9], [10], [11], [12], [13], [14], [15], [16], [17], [18], [19], [20]].

Authors	Age	Gender	Presentation	Joint involved	Association/background
Beltraminellet al. [5]	15	M	Swelling and thickening of the third and fourth digits of the right hand	PIP only	Repetitive rubbing movements of the hands
Al Hammadi et al. [2]	15	M	Non-tender nodules on 2nd to 4th right and left PIP joints	PIP only	No such background except guitar playing
Higuchi et al. [7]	13	F	Symmetrical swelling and thickening of the 2nd to 5th PIP joints of both hands with a small degree of pain	PIP only	No background
Sandobal et al. [8]	Median age of 4 patients: 12 years	3 M, 1 F	2nd to 4th swelling of PIP joints of both hands	PIP only	2 patients were into professional sports and martial arts
Small et al. [9]	12	M	2nd and 3rd digits of left hand demonstrated swelling of PIP joints with evidence of blistering but no pain	PIP of left hand only	Excessive use of gaming consoles, resulting in constant rubbing
Nicolay et al. [10]	14	M	Swelling of 3rd to 5th digits along with thickening of hypothenar and lateral areas of left hand	PIP joints of left hand	Repetitive rubbing was observed as a means of expression
Carrascosa et al. [4]	19	M	Symmetrical swelling of the 2nd to 5th PIP joints laterally	PIP joints	Interlocking of fingers observed and patient had GAD with multiple tics noted on examination
Barnes et al. [1]	25	M	Stiffness and swelling of 2nd to 4th digits laterally on both hands at the PIP and DIP joints as well	PIP and DIP joints	HLA-B27 positive
Chu et al. [6]	17	M	Thickening and swelling of all digits of both hands but 2nd to 4th digits were most prominent	PIP joints only	No background
Iraci et al. [11]	19	M	Swelling of the PIP joints of the 1st to 4th digits with only lateral swelling observed in the first and fourth digits	PIP joints only	No background mentioned
Meunier et al. [12]	Case 1: 18, Case 2: 26	M	Case 1: Progressive swelling of 2nd-3rd PIP joints of both hands with thickening on the sides of the affected joints. Case 2: Swelling on the sides of the PIP joints of the 3rd to 4th digits with thickened skin observed in that area as well	PIP joints only	Both confessed to constant rubbing of fingers
Curley et al. [13]	Mean age of 4 patients: 20 years	M	Case 1: Progressive swelling of PIP joints of 1st to 3rd digits. Case 2: Swelling in PIP joints of both hands with associated discomfort as well. Case 3: Swelling of PIP joints of 1st to third digits. Case 4: Swelling of 1st to third PIP joints of both hands.	PIP only	One of the patients was a labourer and one was a data processor. All 4 denied any family history.
Bardazzi et al. [14]	Mean age of 2 patients: 10 years	F	Case 1: Asymptomatic swelling of 2nd to 4th digits of both hands. Case 2: Swelling of PIP joints of 2nd to 5th digits that was painless.	PIP joints only	One of the patients had a mental retardation disorder (Case 1) and Case 2 had EDS type III with hyperextensibility of the joints observed.
Plana Pla et al. [15]	15	M	Thickening and swelling of 2nd to 4th PIP joints of both hands	PIP joints only	Weight training at a gym, 4 times a week
Woodrow et al. [16]	15	M	Soft tissue thickening and swelling of 2nd to 4th PIP joints of both hands	PIP joints only	Asperger sydrome with history of repetitive manipulations of the hands
Sagransky et al. [17]	Mean age of 2 patients: 38 years	M	Case 1: Swelling of the lateral sides of the 2nd to 3rd PIP joints of both hands with pain and itching. Case 2: Pain and itching with swelling of 2nd to 3rd PIP joints.	PIP joints only	Both worked in poultry processing which involves constant repetitive movements of the hands. Case 1 was a chicken catcher and Case 2 was a chicken hanger. Both reported a decrease in symptoms on taking days off from work.
Seo et al. [18]	14	M	Bilateral swelling of 2nd to 4th PIP joints	PIP joints only	Demonstrated repetitive movements of the hands and had been sent for an emotional consultation to a psychologist in the past
Bardazzi et al. [19]	23	F	Painful fusiform swelling of the 4th PIP on right hand (Swelling localized on ventral, dorsal and lateral sites of the finger)	PIP of right hand only	Painful involvement and limited to one finger only (4th PIP of right hand)
Russo et al. [20]	Case 1: 28, Case 2: 61	F	Case 1: Bilateral swelling of 4th and 5th PIP and DIP of right hand and 2nd and 3rd PIP and DIP of left hand. Case 2: Bilateral swelling of PIP and DIP on both hands and 5th digit revealed a subcutaneous nodule	PIP and DIP joints	First documented case of familial pachydermodactyly (Mother and Daughter)

## 4 Discussion

The classical presentation of PDD is a bilateral, symmetrical, progressively painless and non-pruritic thickening of the lateral aspects of the second through fourth PIP joints of the hand, resulting in a fusiform or saccular swelling pattern, while typically sparing the thumb [3]. Other patterns including unilateral, localized known as monopachydermodactyly (affecting a single joint), and transgressing (extending to the dorsum of hand and metacarpophalangeal joints) have been previously reported in the literature [9,10,19]. Laboratory investigations, including inflammatory markers erythrocyte sedimentation rate (ESR) and C-reactive protein (CRP), ANA, serum RF, and thyroid function tests, are normal in all reported cases. Radiological imaging reveals soft tissue swelling in the absence of bony or articular manifestations including synovitis, joint effusions, erosions, cysts and osteophytes [3]. Histopathologic findings are non-specific and similar to other fibromatoses, and are only useful in ruling out other entities on the differential diagnosis. For this reason, histopathologic examination is not required for the diagnosis of PDD [8]. These findings include an acanthotic epidermis with compact hyperkeratosis, and a thickened dermis with haphazardly arranged coarse collagen bundles, slight proliferation of fibroblasts, with no inflammatory infiltrate [1,2].

PDD is thought to be a result of excessive mechanical manipulation of the PIP joints, usually by repetitive minor trauma involving interlacing the fingers and repeated rubbing of the fingers [18]. It has been associated with OCD, GAD, various athletic and occupational activities that subject individuals to repetitive skin trauma, Asperger syndrome, and Ehlers-Danlos syndrome [4,5,11,14,16,17]. Russo et al. have reported a rare case of familial pachydermodactyly [20]. The symptomatic form of PDD, which may be painful, has been linked to tuberous sclerosis [14]. However, our patient presents with the symptomatic painful form of PDD, despite having no significant past medical history.

The diagnosis of PDD is mainly clinical, and requires a high index of suspicion after ruling out all other causes of PIP joint swelling. PDD presents a diagnostic challenge due to its ability to mimic several other etiologies that render the same physical finding of progressive PIP joint swelling. When clinical acumen has been excised to rule out most other potential differential diagnoses and there is an active suspicion for PDD, the absence of three clinical indications becomes very useful in assessing whether clinicians should pursue the possibility of PDD further or not. This triad includes the absence of pain, tenderness and reduced functionality at the joints involved. Additionally, these three clinical features are also of benefit when viewing the other possible causes or ruling out PDD earlier on. A background of occupational exposure such as poultry processing or prior psychiatric diagnoses such as of OCD or GAD in addition to the aforementioned clinical findings is what usually necessitates a diagnosis of PDD. Furthermore, a thorough physical examination, and radiographic studies are required to distinguish PDD from other cases of PIP joint swelling (Table 2) particularly rheumatoid arthritis, juvenile chronic arthritis, or psoriatic arthritis [3].

**Table 2:** A tabulation of the possible differential diagnoses.

Diagnosis	Clinical signs and symptoms
Pachydermodactyly	No pain, swelling, tenderness, loss of range of motion. Does not itch generally and bilateral involvement.
Inflammatory arthritis: Psoriatic arthritis, Rheumatoid arthritis (RA), Juvenile idiopathic arthritis	Pain, tenderness, decreased range of motion.
Mechanical etiology: Osteoarthritis	Pain, decreased range of motion, stiffness.
Knuckle pads/Garrod's pads	Skin-colored subcutaneous nodules.
Pseudoknuckle pads	Similar to knuckle pads.
Lichen Simplex Chronicus	Usually itchy with only slight swelling present.
Self-healing juvenile cutaneous mucinosis	Papules on various parts of the body.
Early systemic sclerosis	Diffuse swelling of digits. Late cases show ulceration and telangiectasias.
Dermatomyositis	Inflammatory papules of scaly appearance.
Acanthosis nigricans	Plaques that have a hyperpigmented, velvety appearance.
Thiemann's disease	Shortening of affected PIP joints with shortening of digits. Pain may be present.
Systemic Lupus Erythematosus Dermatitis	Erythema, dorsal involvement of the digits with sparing of the knuckles.
Mixed Connective Tissue Disease	Digits of the hand appear swollen or sclerosed with evidence of Raynaud's phenomenon and arthritis. Cutaneous lesions might also be present.
Referred pain due to cervical spondylosis	Pain in the neck can spread to the hands as well. Additionally, weakness and numbness of the hands may also be present.
Benign tumors such as fibromas	Presentation may vary.
Tuberous sclerosis	Swelling.
Primary pachydermoperiostosis (Touraine-Solente-Golé syndrome)	Periarticular tissue proliferation with clubbing evident.

Treatment of PPD is not typically required due to the benign course of the disease. In certain cases, where repetitive trauma is thought to be the cause, behavioral modification and counseling may lead to gradual resolution [1]. In cases where PDD is thought to be as a result of OCD, or GAD, patients might benefit in treatment of the underlying cause, and a psychotherapy referral might be warranted. While topical corticosteroids are ineffective, treatment with intralesional triamcinolone has been documented to result in a reduction of the degree of swelling [12,13,15]. In selected cases, surgical excision might be an effective cosmetic option [8]. Higuchi and colleagues reported the use of tranilast, an inhibitor of collagen synthesis in human skin fibroblasts, commonly used as an antiallergic drug, displaying reduction in the degree of swelling [7].

## 5 Conclusion

Despite the undeniable predilection toward affecting younger populations, pachydermodactyly can also afflict adults. It is due to the benign, asymptomatic course of PDD that delays in seeking medical intervention persist. It is therefore evident that PDD, although non-sinister in nature, is easily capable of mimicking a vast array of etiologies underlying swelling of the joints of the hands.

## Funding

None.

## Availability of data and material

All data shown.

## Code availability

Not applicable.

## Guarantor

Salman Hussain.

## Authors’ contributions

SH: conducted literature review, drafted the manuscript and critically revised it. ME: conducted literature review and drafted the manuscript. TA: drafted the manuscript and critically revised it. AA: diagnosed the case and critically revised manuscript. All authors approved the final manuscript as submitted and agreed to be accountable for all aspects of the work.

## Ethics approval

This case is compliant with the ethical standards of the institutional and/or national research committee and with the 1964 Declaration of Helsinki and its later amendments or comparable ethical standards.

## Consent to participate

Informed consent was obtained by the patient.

## Consent for publication

Informed consent was obtained by the patient.

## Declaration of competing interest

None.
